# Income Disparities and the Global Distribution of Intensively Farmed Chicken and Pigs

**DOI:** 10.1371/journal.pone.0133381

**Published:** 2015-07-31

**Authors:** Marius Gilbert, Giulia Conchedda, Thomas P. Van Boeckel, Giuseppina Cinardi, Catherine Linard, Gaëlle Nicolas, Weerapong Thanapongtharm, Laura D'Aietti, William Wint, Scott H. Newman, Timothy P. Robinson

**Affiliations:** 1 Biological Control and Spatial Ecology, Université Libre de Bruxelles, Brussels, Belgium; 2 Fonds National de la Recherche Scientifique, Brussels, Belgium; 3 Animal Production and Health Division (AGA), Food and Agriculture Organization of the United Nations (FAO), Rome, Italy; 4 Department of Ecology and Evolutionary Biology, Princeton University, Guyot Hall, Princeton, New Jersey, United States of America; 5 Department of Livestock Development, Bangkok, Thailand; 6 Environmental Research Group Oxford, Department of Zoology, Oxford, United Kingdom; 7 Emergency Center for the Control of Transboundary Animal Diseases (ECTAD), Food and Agriculture Organization of the United Nations (FAO), Hanoi, Vietnam; 8 Livestock Systems and Environment (LSE), International Livestock Research Institute (ILRI), Nairobi, Kenya; CEFE, FRANCE

## Abstract

The rapid transformation of the livestock sector in recent decades brought concerns on its impact on greenhouse gas emissions, disruptions to nitrogen and phosphorous cycles and on land use change, particularly deforestation for production of feed crops. Animal and human health are increasingly interlinked through emerging infectious diseases, zoonoses, and antimicrobial resistance. In many developing countries, the rapidity of change has also had social impacts with increased risk of marginalisation of smallholder farmers. However, both the impacts and benefits of livestock farming often differ between extensive (backyard farming mostly for home-consumption) and intensive, commercial production systems (larger herd or flock size, higher investments in inputs, a tendency towards market-orientation). A density of 10,000 chickens per km^2^ has different environmental, epidemiological and societal implications if these birds are raised by 1,000 individual households or in a single industrial unit. Here, we introduce a novel relationship that links the national proportion of extensively raised animals to the gross domestic product (GDP) per capita (in purchasing power parity). This relationship is modelled and used together with the global distribution of rural population to disaggregate existing 10 km resolution global maps of chicken and pig distributions into extensive and intensive systems. Our results highlight countries and regions where extensive and intensive chicken and pig production systems are most important. We discuss the sources of uncertainties, the modelling assumptions and ways in which this approach could be developed to forecast future trajectories of intensification.

## Introduction

Livestock today represent the largest biomass of terrestrial vertebrate species [1,2]. This is a consequence of rapid changes in human demography and eating habits, which have led to substantial increases in livestock production. These have accelerated during the last few decades; a period sometimes referred to as the livestock revolution [3,4], which has been characterized by i) shifts to vertically-integrated, market-oriented production, ii) a decreasing importance of ruminants in comparison to monogastric species, such as pigs and poultry, iii) large-scale industrial production closer to urban consumption centers, iv) an increase in the use of cereal-based feed, and v) an increase in the global volume of trade in live animals and livestock products [3,4]. The increase in stocks and productivity has been particularly marked for monogastric species, most especially chickens and pigs. Between 1960 and 2010, the global stocks of chickens and pigs increased by factors of 5 and 2.5, respectively [5]. During the same period, annual meat output per animal increased from 1.7 to 4.0 kg per bird for chickens, and from 60.9 to 111.2 kg per head for pigs. Combining those estimates of stocks and productivity, global production of chicken and pig meat increased by factors of 11.5 and 4.3, respectively over a period of 50 years. During the same period, the human population rose only by a factor of 2.3, from 3.0 to 6.9 billion [6]: a comparatively modest increase. This massive growth in chicken and pig meat production has been achieved largely by intensifying production through increasing animal densities, constructing larger production units, increasing efficiency of the processing infrastructure, using more concentrated feeds, increasing mechanization and the widespread use of pharmaceuticals such as antimicrobials and vaccinations [4].

However, the proportion of animals raised in intensive production systems is extremely variable between countries, depending largely on their levels of economic development, and this represents an important distinction to make in order to understand the impacts and benefits of livestock production.

In high-income countries, the overwhelming majority of chicken and pig production is intensive, taking advantage of the high levels of inputs to optimize feed conversion ratios. Only a small proportion of chickens and pigs is raised under extensive production systems and, while demand for organic farming products is increasing faster than the average growth of the agricultural sector, it still remains low in absolute terms [7]. This leads to the paradox that countries like Belgium and the Netherlands have some of the highest densities of chickens and pigs per km^2^ in the world, yet these are hardly seen in the landscape as they are hidden away in intensive production units. The situation differs greatly in low-income countries, where the vast majority of chickens and pigs is raised under extensive conditions by family-based smallholder farms. In transition economies, extensive backyard production co-exists with intensive farming, to the extent that a very clear bimodal distribution of flock or herd size per holder can be observed, as for example in Thailand [8].

The process of intensification, by which extensive production systems, with low input and outputs, are replaced by more specialized intensive farms, has been particularly rapid in much of Southeast Asia, but with contrasting levels across the continent. For instance, whilst 75–90% of poultry are raised in extensive backyard systems in countries such as Bangladesh, Cambodia, India, Myanmar or Nepal, only 30% were produced in extensive systems in Thailand in 2005, and less than 5% in countries such as Malaysia, Japan and South Korea (see methods for sources). Because demographic growth and increasing wealth are pushing up levels of consumption of animal-source foods, demand for poultry meat is forecast to increase rapidly in Asia in the coming decades. It has been estimated that growth in demand for poultry meat from 2000 to 2030 will be around 121% in China and a staggering 844% in India [9], although a recent revision of FAO’s projections suggests slightly less dramatic figures for poultry growth in India [10]. Accompanying such changes will be a transformation of market structures towards networks with fewer larger markets where an increasing volume of products will be traded.

The intensification of poultry and pig production is absolutely necessary to meet demands of a growing and more affluent population, but it comes, potentially, with significant environmental and health risks. There are three areas of particular concern. First, the concentration of very high number of animals on small areas of land creates manure management problems because of the lack of sufficient cropland surface to permit efficient natural nutrient recycling. As a result, intensive poultry and pig production is frequently associated with localised pollution of land and water resources [11]. Second, a combination of factors make animals raised in intensive production systems particularly vulnerable to pathogens and environmental disturbance (temperature, air recycling). The breeds raised have mostly been selected for their production characteristics, rarely for their resistance to diseases, and the high densities facilitate the transmission of diseases. As a consequence, intensive poultry and pig production requires the extensive use of preventive and curative pharmaceuticals. Antimicrobials are used in abundance, not only for prophylaxis and treatment, but also as a feed additives for growth promotion, with an estimated total of 63,153 tons of antibiotics consumed by livestock globally in 2010 [12]. Whilst the regulation of antimicrobials at sub-therapeutic levels for growth promotion is imposed in many countries, and recent data question the cost-effectiveness of such practices [13–15], it is of growing concern in some of the major emerging economies of the world. The abuse of antimicrobials in livestock production creates ideal conditions for the emergence of antimicrobial resistance in pathogens, which is becoming an issue of global importance [12,16], particularly given the speed with which modern transportation networks can spread such antimicrobial-resistant pathogens around the World [17]. Third, the conditions encountered in intensive poultry and pig production systems may favor the evolution of more virulent forms of pathogens, particularly viruses [18–20], through a lower cost (to the pathogen) of virulence associated with high densities and genetic homogeneity. Highly virulent forms of several pathogens have emerged in regions dominated by intensive or intensifying commercial systems, such as highly pathogenic avian influenza viruses (e.g., H7N7 in the Netherlands [21], H7N1 in northern Italy [22] and H5N1 in China [23]), and the highly pathogenic porcine respiratory and reproductive virus variant (PRRS) [24]. Making the distinction between production systems is therefore essential for epidemiological applications. In spite of the obvious need to account for these differences such a distinction has rarely been possible in epidemiological analyses, as illustrated in a risk factor meta-analysis of HPAI H5N1 investigations [25], though notable exceptions do exist [26].

As well as environmental and health implications there may be important social consequences of production intensification, but these are much less well understood. Whilst there may be positive aspects such as a greater availability of affordable, safe protein, the quality of meat may be lower from intensive production systems in which animals are pumped with growth promoters and antibiotics. Similarly, whilst intensive production and associated food systems may provide important employment opportunities, it may also squeeze less competitive, smaller producers out of markets. Whether the net social impacts of intensification are positive or negative needs investigation and is likely to vary considerably in different contexts [27–29].

So, mapping the geographical distribution of poultry and pig production systems is likely to be extremely important in understanding the environmental, social and health impacts of intensification of livestock production. Several previous studies, have developed methods to map different species of livestock [30–33], and existing maps of the global livestock distribution have recently been revised, using new methodologies and data [34]. However, very little work has yet been done to separate extensive from intensive production in distribution maps.

This study addressed this shortfall by taking two important steps subsequent to the modelling of livestock densities. In the first step we develop a new approach whereby the proportion of extensively raised chicken or pigs is modelled as a function of gross domestic product (GDP) per capita (in purchasing power parity for 2010 (PPP_2010_)), based on the following assumptions. The consumption demand for animal products per capita, expressed in kg of meat consumed per person per year, was previously shown to be strongly linked to GDP per capita [35]. So, increasing incomes creates higher demand for animal products and this represents a strong economic incentive for investments in intensification. A corollary is that the investment needed by a producer to intensify its production units may also be more easily found in an economy with rising income. Agricultural development also largely contributes to rising GDP, especially in poor and transition economies, which further supports the links between intensive production and GDP. Given the size of China compared to other countries, and the strong differences in economic development and policies for different provinces, the GDP model was developed and applied at the country-level in all countries except in China where province-level data were used. We then used this model to predict the proportion of extensively raised animals in countries where the figure was not found through data-mining and to distribute the estimated number of extensively raised animals as a function of the human rural population, estimating commercially raised chickens by difference from the total, and adjusting for the rare pixels where the predicted number of extensively raised chickens exceeded the total number of predicted chickens.

## Materials and Methods

### Data

A variety of country and province level figures were extracted from publicly available databases. The production of meat (metric tons per year) from pigs and poultry, as well as their respective stocking rates were obtained from FAOSTAT [5]. National Gross Domestic Product (GDP) estimates, in purchasing power parity (PPP), were obtained by country from the World Bank database [36] for the reference year 2010, and for the years for which matching poultry and pig production system figures were available. GDP in PPP was used instead of raw GDP as it corrects for purchasing power disparities between countries, and is therefore more suitable for country-to-country comparisons of GDP per capita. For China, sub-national province GDP estimates (in purchasing power parity) were obtained from the China National Bureau of Statistics for the year 2010 [37]. The proportions of chickens raised under extensive backyard production systems (*P*_*ext*_) were data-mined for a number of countries from various literature sources, with a definition of backyard production systems following that of FAO sector 4, i.e. village or backyard production with minimal biosecurity and birds and products being consumed locally [38]. For pigs, a similar assessment was made, but distinguishing extensive (*P*_*ext*_), semi-intensive (*P*_*sint*_) and intensive (*P*_*int*_) production systems, according to the definition set out in Robinson *et al*. [35]:

- Extensive: Usually unconfined. Typically < 10 pigs. Scavenging supplemented with household waste. Virtually no use of feed that is purchased or grown specifically as feed. Non-descripted or local breeds. Little or no prophylactic or remedial health care. Family consumption with occasional local marketing on an ad hoc basis. Usually not specialised.
- Semi-intensive: Usually confined, sometimes with partial scavenging. Typically 10–100 pigs. Home-produced or collected feeds with, in some cases, purchased supplements. Various breeds from non-descript / local through crossbreds to specialized breeds suitable for the relatively extensive production conditions. Improved monitoring of health status with some use of vaccination / medication for locally significant ailments. Some family consumption / ad hoc marketing may still take place but more likely to access defined, local markets. Often specialised into piglet production or fattening units.
- Intensive: Largely confined, although intensive outdoor management is widely practiced in some parts of the World. Typically > 100 pigs. Some feed inputs (grains generally) may be home produced but significant purchases either as straights or ready-mixed. Highly-bred, specialist stock. Routine, vaccination, preventative and remedial health care with advanced health management (e.g. quarantine, culling). All stock marketed through various arrangements including contract rearing. Specialised into piglet production or fattening units.

Data on the proportion of stock raised under different systems were found for 86 and 97 countries for chicken and pigs, respectively (S1 Fig).

The analyses also required three spatial databases. First, we used the most recent revisions of the pig and chicken distribution maps from the gridded livestock of the world (GLW 2.0) published by Robinson *et al*. [34] and aggregated these data at a spatial resolution of 5 minutes of arc (0.083333 decimal degrees, or approximately 8 × 8 km at the equator) to obtain the total number of chickens or pigs per pixel. This data set was also used to estimate the total stocks of chickens and pigs per province in China. Second, the human population data layer was obtained from the 2010 version of the LandScan dataset [39] adjusted with country values to 2010 United Nations (UN) estimates [6]. These data were aggregated at the same spatial resolution of 5 minutes of arc to obtain the total human population per pixel and corrected to match FAOSTAT 2010 country totals. Finally, in order to remain consistent with the GLW 2.0, the same masking criteria were used to exclude land pixels defined as unsuitable for chicken and pig production, i.e. with elevations higher than 4,750 m above sea level, at a slope of gradient higher than 40 percent, urban areas and pixels permanently covered with snow or ice [34].

### Analysis

The overall analysis pathway involved five steps: i) creating a model relating the proportion of extensively raised animals (chicken or pigs) at the national level to GDP per capita (in purchasing power parity for 2010 (PPP_2010_)), ii) using this model to predict the proportion of extensively raised animals in countries where the figure was not found through data-mining, iii) distributing the estimated number of extensively raised animals equally among the rural human population, iv) estimating commercially raised chickens by difference from the total, and v) adjusting for the rare pixels where the predicted number of extensively raised chickens exceeded the total number of predicted chickens. For pigs, the procedure included an extra step to separate the semi-intensive category.

First, we modelled the national or province-level proportion of extensively raised chickens or pigs obtained by data-mining as function of GDP per capita (PPP_2010_). In order to bound the predictions between 0 and 1, we used a logistic model where the dependent variable was modelled as:

$$P_{ext}\;=\;1/\left(1+e^{\left(4.\mu_{ext}.\left(\lambda_{ext}-GDP_{PPP}\right)+2\right)}\right)$$

where *P*_*ext*_ is the proportion of extensively raised chickens, *GDP*_*PPP*_ is the log10-scale GDP per capita of the country (PPP_2010_), and *μ*_*ext*_ and *λ*_*ext*_ parameters of the model controlling, respectively, the steepness of the growth and its position. *P*_*int*_ for chicken was simply estimated by default as 1- *P*_*ext*_. For extensively and intensively raised pigs, we used the following models:

$$P_{ext}\;=\;\alpha_{ext}/\left(1+e^{\left(4.(\mu_{ext}/\alpha_{ext}).\left(\lambda_{ext}-GDP_{PPP}\right)+2\right)}\right)$$

$$P_{int}\;=\;1-\;\alpha_{int}/\left(1+e^{\left(4.(\mu_{int}/\alpha_{int}).\left(\lambda_{int}-GDP_{PPP}\right)+2\right)}\right)$$

where all terms are defined as above, except *P*_*int*_, the proportion of pigs raised under intensive systems, and *P*_*ext*_ and *P*_*int*_, additional model parameters used to account for the maximum proportion of *P*_*ext*_ and *P*_*int*_ that may, in this case, differ from 1. *P*_*sint*_ was simply estimated as 1—(*P*_*ext*_ + *P*_*int*_). The model coefficients were estimated using non-linear least squares regression. In order simultaneously to account for the variability in the observed data and for the different stocking levels in different countries we used a Monte Carlo simulation where we sampled 1,000 times 25 countries out of the observed dataset with replacement, with a probability of being in the sample estimated as the ratio of the national livestock population of chickens or pigs to the global total for that species. This effectively weighted the contribution of data points to the analysis by population, ensuring that countries with high chicken or pig populations would be more often selected in the sample.

Second, the model was used to predict *P*_*ext*_ and *P*_*int*_ in all countries and Chinese provinces where no observed value had been found through data-mining, using the median predicted value of the 1,000 bootstraps. For countries with observed values, the observed value was retained if it was within the 1%- 99% percentile range of the 1,000 bootstrap predicted values. If the observed value was outside this range, the upper (99% percentile), or lower (1% percentile) predicted value was used. This allowed most extreme values in the observed data set to be filtered out and these were mainly from unofficial sources such as personal communications. This resulted in predicted or observed values of the proportions of chickens (*P*_*ext*_ + *P*_*int*_) and pigs (*P*_*ext*_, *P*_*sint*_, *P*_*int*_) in each system for all 205 countries, and an accompanying value for the total stocks of chickens and pigs from FAOSTAT 2010.

Third, the proportions of chickens (*P*_*ext*_ + *P*_*int*_) and pigs (*P*_*ext*_, *P*_*sint*_, *P*_*int*_) in each system for all countries and provinces were used in combination with maps of rural population to map chickens and pigs global, by system. The predicted proportions of extensively raised chickens and pigs were multiplied by the numbers of chickens and pigs in each country and Chinese provinces in 2010 to the number of extensively raised chickens or pigs for each spatial unit. Within each unit, those numbers were then distributed equally among the rural population. The total rural population for each country or Chinese province was estimated by summing the values for all pixels in the rural population GIS layer, having masked for monogastric suitability. These values were used to estimate the number of extensively raised chicken and pigs per rural person in areas suitable for production. These national or provincial multipliers were then applied to the raster layer of rural population for each country to derive the numbers of extensively raised chickens and pigs per pixel.

Fourth, commercially raised chickens (*P*_*int*_) and pigs (*P*_*sint*_ + *P*_*int*_) in each pixel were then estimated by subtracting the number of extensively raised chickens or pigs from the total number of chicken or pigs per pixel from GLW 2.0.

Fifth, in some instances, the number of extensively raised chicken or pigs per pixel exceeded the total value predicted by GLW 2.0. In that case, the GLW 2.0 value was replaced by the number of extensively raised animals, and the difference in numbers was summed over the entire country or province, and subtracted pro-rata from each pixel within the spatial unit from the commercially raised chickens or pigs, to ensure that the total stock remained unchanged when summed at the country or province level over all systems.

Finally, the pixel-level estimates of intensively raised pigs (*P*_*sint*_ + *P*_*int*_) were divided into pixel-level *P*_*sint*_ (semi-intensive) and *P*_*int*_ (intensive) distributions according to their national or province-level observed or predicted ratios, the assumption being that semi-intensively raised pigs do not necessarily follow rural population.

## Results

**Fig 1:**
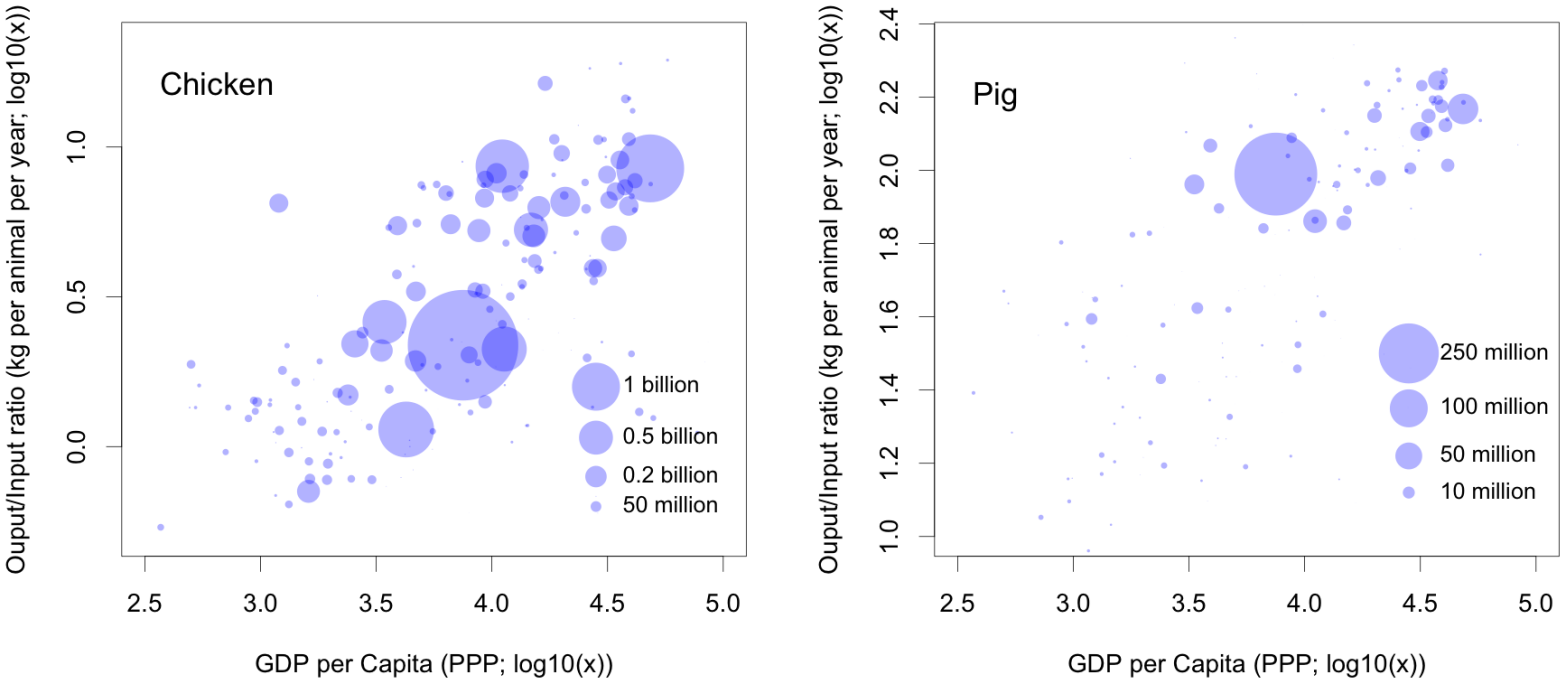
Productivity (kg of meat per animal per year) as a function of Gross Domestic Product per capita (USD in purchasing power parity in 2010). Each dot represents a country, with the size indicative of the stock according to FAOSTAT [5]. Only countries with stocks > 0 for chickens (*n* = 190) and pigs (*n* = 170) are included.

**Fig 2:**
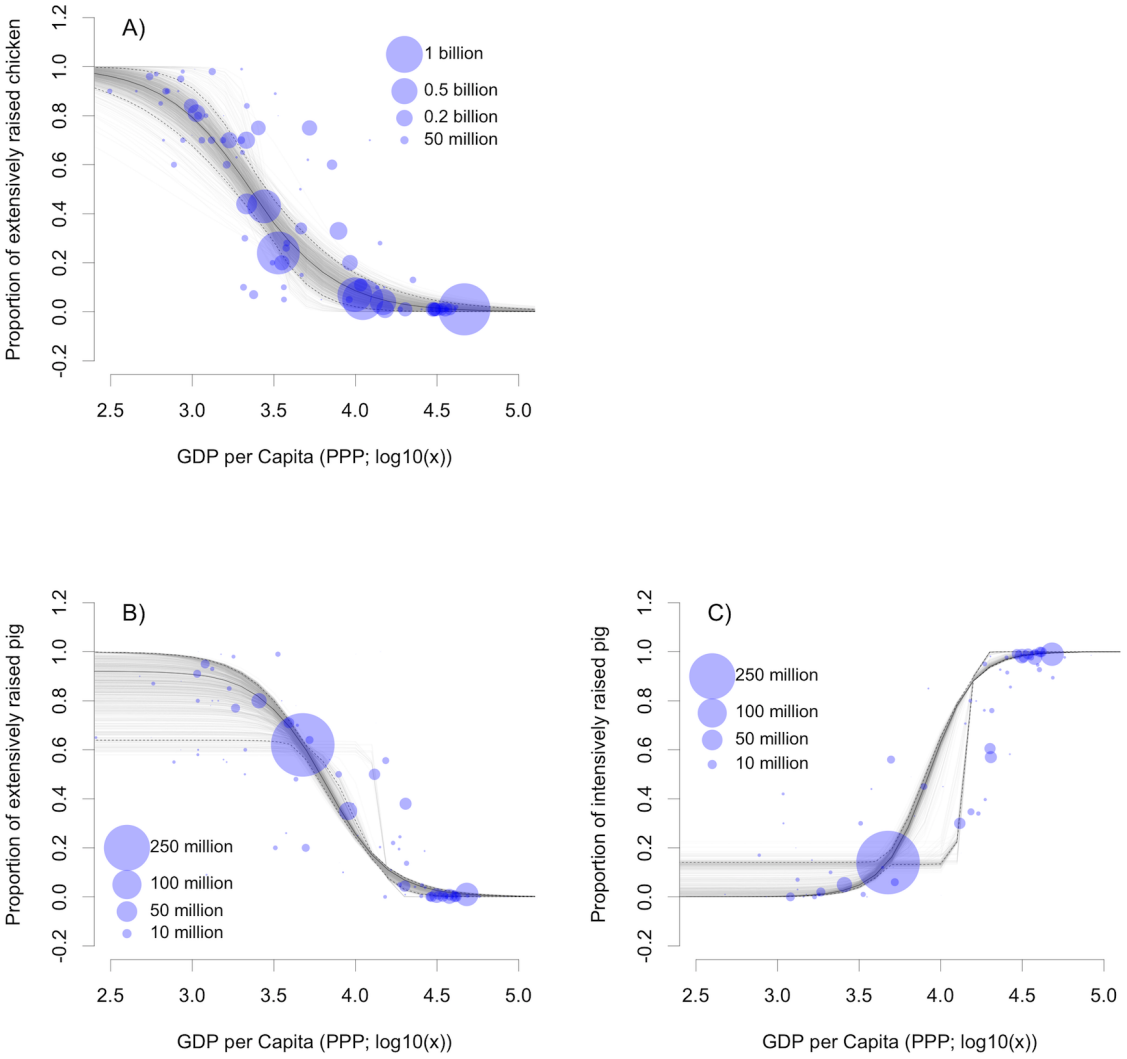
Models of the proportions of extensively raised chickens (a) and pigs (b) and of intensively-raised pigs (c) as a function of GDP per capita (USD, Purchasing Power Parity for 2010). Each dot represents a country for which *P*_*ext*_ and *P*_*int*_ was established through data-mining (*n* = 86 for chicken, *n* = 97 for pig), with the size indicative of the stock according to FAOSTAT [5].

**Fig 3:**
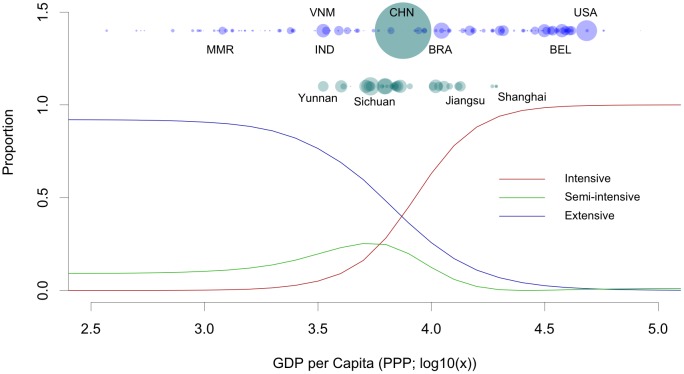
Predicted proportions of pigs raised under extensive, semi-intensive and intensive production systems as a function of GDP per capita (USD, Purchasing Power Parity). The top row of points indicates the position of different countries along the gradient of GDP per capita, with the size of the points indicative of the national stock according to FAOSTAT [5]. A selection of countries is indicated by their ISO-3 codes.

GDP per capita is correlated with productivity, measured in terms of kg of product per animal per year, for both chickens and pigs (Fig 1). Intensification of agricultural production in general is both a cause and a consequence of increasing wealth. Agriculture contributes to economic development and, in turn, benefits from the higher demands for animal products, which, with a few cultural exceptions, are associated with increasing wealth. This relationship underpins the skill with which GDP per capita (in Purchasing Power Parity, PPP) can predict the proportion of extensively raised chickens and pigs (Fig 2); with correlation coefficients of 0.856 and 0.848 between observed and predicted values for chickens and pigs, respectively. This relationship also allows the proportion of intensively raised pigs to be predicted with similar levels of accuracy (r = 0.878), and allows inferences to be made as to how the modeled proportion of pigs raised under extensive, semi-intensive, and intensive production systems vary with increasing wealth at the country or province level (Fig 3). Below 1,000 USD per capita, over 90% of chicken are raised under extensive systems and the transition from extensive to intensive production really occurs between 1,000 and 10,000 USD per capita; above which most chickens are raised in intensive systems. For pigs, the transition zone—within which pigs are raised under a mixture of extensive, semi-intensive and intensive systems—extends between 1,000 and 30,000 USD per capita. Countries with per capita GDP levels in excess of 30,000 USD tend to raise more than 95% of their pigs in intensive systems.

**Fig 4:**
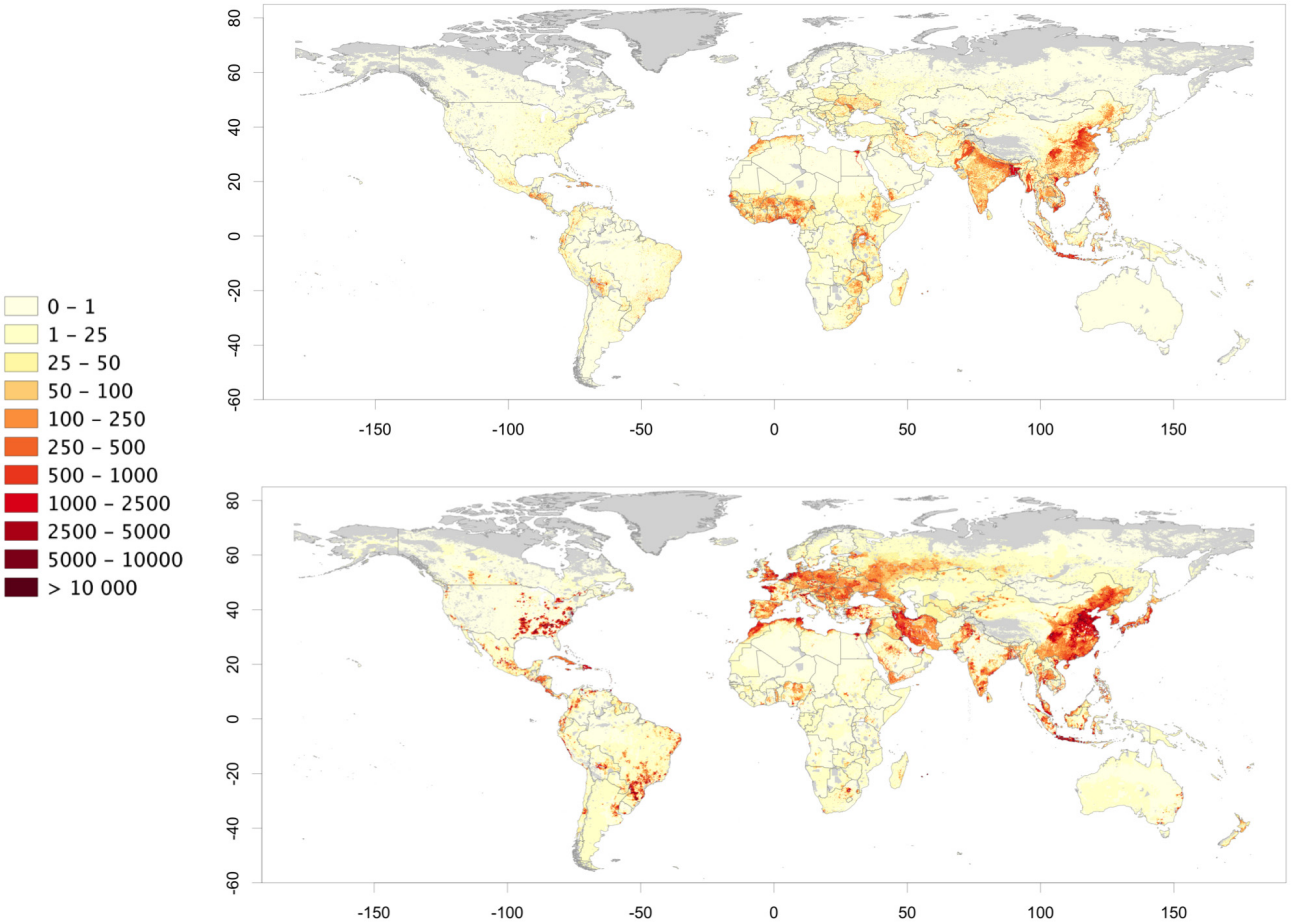
Distribution of chickens (birds per square kilometre) raised under extensive (a) and intensive (b) production systems (unprojected lat/long decimal degrees coordinate system, WGS 84). The data used to produce these maps were all from public sources (detailed in the Material and Method section), and the country limit data are from the FAO Global Administrative Unit Layers (GAUL) database.

When these estimates are translated into maps, contrasting patterns become apparent for extensive and intensive chicken and pig production systems. For chickens, the highest densities of extensively raised birds are found in south, east and southeast Asia, in several highly populated countries of Africa and in parts of Central America and the Caribbean (Fig 4). Hotspots of high animal densities are found in Bangladesh, in the Vietnam deltas, in the island of Java in Indonesia, and in the Nile delta. In absolute terms, China, India, Indonesia, Bangladesh, Pakistan and Nigeria represent the countries with the greatest populations of extensively raised chickens, between them accounting for more than half of all extensively raised chickens in the World. The highest densities of intensively raised chickens are found in southeast United States, west Europe, south Brazil, south India and northeast China. China accounts for 27.6% of the global population of intensively raised chickens, followed by the United States (11.8%), Brazil (7.0%) and Indonesia (6.1%). China and Indonesia are thus listed in the top 5 countries for both extensively and intensively raised chickens.

**Fig 5:**
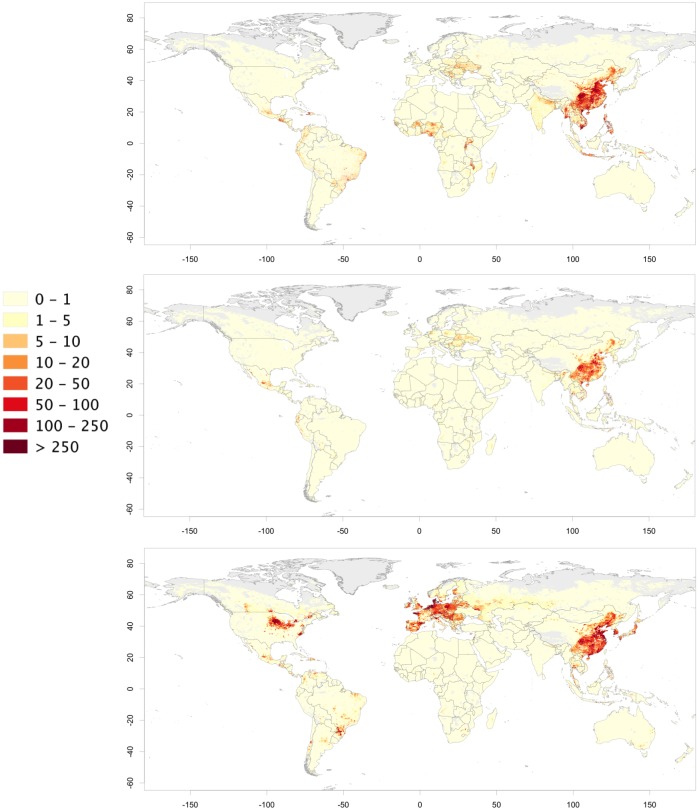
Distribution of pigs (head per square kilometre) raised under extensive (a), semi-intensive (b) and intensive (c) production systems (unprojected lat/long decimal degrees coordinate system, WGS 84). The data used to produce these maps were all from public sources (detailed in the Material and Method section), and the country limit data are from the FAO Global Administrative Unit Layers (GAUL) database.

The highest densities of pigs raised in extensive systems occur in Asian countries (Fig 5), with China alone accounting for 69.1% of the global total, followed by Vietnam (5.3%), Brazil (3.3%), Philippines (2.3%) and Myanmar (2.1%). Outside Asia, large populations of extensively raised pigs are found in Latin America, in a few African countries and in east European countries such as Ukraine, but their densities and numbers by no means compare to those of Asia and China. Intensively raised pigs are found in highest densities in Europe, in the United States Midwest, in southern Brazil, China, Japan and South Korea. China stands out with the highest proportion of the global total (16.8%), on a par with the United States (16.2%), and followed by Germany (6.6%), Brazil (6.4%) and Spain (6.3%).

**Fig 6:**
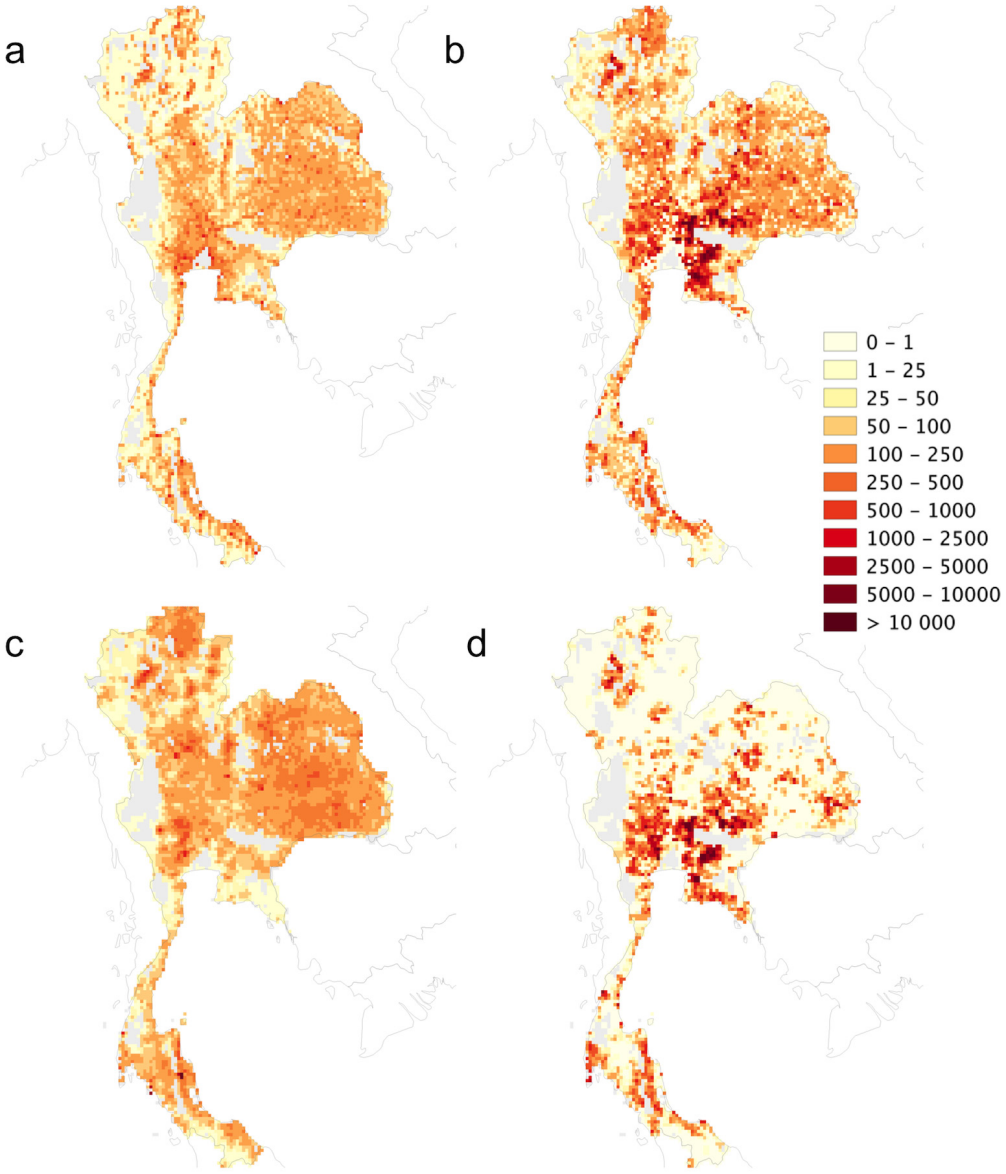
Extensively and intensively raised chickens in Thailand according to the current methodology (a and b, respectively) and that of Van Boeckel *et al*. [8] (c and d, respectively) (unprojected lat/long decimal degrees coordinate system, WGS 84). The data used to produce these maps were all from public sources (detailed in the Material and Method section) and the country limit data are from the FAO Global Administrative Unit Layers (GAUL) database.

The results of the present study have been compared to those of Van Boeckel *et al*. [8] based on extremely detailed census data in Thailand that allowed the fine-scale distribution of chickens raised under extensive and intensive systems to be mapped (Fig 6). The figure illustrates that the two methods obtained very similar patterns of intensive and extensive production. The areas with the highest densities of chickens raised under intensive production systems were well captured in the present analysis though it tends to overestimate the spatial extent of intensively raised chickens in intermediate density classes.

## Discussion

The maps presented here show the global distributions of chickens and pigs by production system helping us to understand their broad patterns and linking those patterns to different environmental, health and social outcomes of monogastric livestock production. For example, whilst it is obvious that Bangladesh has very different chicken farming systems than does northern Europe, it would be far less straightforward to distinguish the situation in Bangladesh from that in some parts of China. The overall chicken density in northeast China is higher than that of Bangladesh but Bangladesh has higher densities of extensively raised chickens than most parts of populated China. The opposite holds true when comparing the densities of intensively raised birds in these two countries. In global terms, chicken production in North and South America and in Europe is dominated by intensive systems. In contrast, extensive chicken production dominates in most of Africa. Asia presents a somewhat intermediate situation with many countries where both extensive and intensive production systems coexist in high densities, reflecting the transitional nature of this region.

For pigs, few countries except China still have high densities of pigs raised in extensive and semi-extensive production systems. China alone accounts for nearly 70% of pigs raised under extensive systems globally. The country is therefore likely to be a major hot-spot in terms of intensification of pig production. If demand were to remain constant then intensification of pig production in China, with increased carcass weights and off-take rates, would result in a reduction of the number of animals required to meet that demand. However, the demand for pig meat is projected to increase by 38%- 54%, depending on sources, between 2000 and 2030, so the gains in efficiency by intensification may not be reflected in a reduction in stock.

The maps of extensively and intensively raised chickens and pigs also highlight strong differences in their spatial patterns; with intensive production being more clustered spatially. For example, the level of clustering of intensive chicken and pig production in the United States, Brazil and northwestern Europe are particularly noticeable. Similar patterns were observed at more local and refined scale in the outputs of the fine-scale census study in Thailand. Some regions become specialized in intensive pig and poultry production and have locally high densities (relative to their human population) than others, mostly influenced by the availability of cheap feed, either locally (e.g. pigs in the United States Midwest), or through ports of entry (e.g. pigs in Brittany, in Flanders and the Netherlands).

As can be seen from Fig 2, many countries are still in transition from extensive to intensive production systems and these include countries with extremely high populations and anticipated relative and absolute increases in demand such as China and India. If these countries follow similar patterns of intensification, one could expect the distribution of intensively raised chickens and pigs to become clustered in high production zones, and the distribution of extensively raised chickens and pigs gradually to fade out. The consequence may be a corresponding change in the distributions of negative and positive impacts. Negative impacts will become much more locally intense. As production of livestock becomes disconnected from the land used to produce the feed-crops, the cycling of nutrients will become increasingly disrupted resulting in nutrient mining from the areas producing the feed (most likely to be replaced by inorganic fertilizers) and nutrient loading in areas of dense, intensive livestock production. Whilst intensification may provide a source of affordable protein to burgeoning urban populations, the benefits of poultry and pig farming will involve fewer, larger farms; smallholder farmers possibly being out-competed in the marketplace. The positive impact will be more broadly distributed according to the distribution of consumers who can benefit from the availability of cheap animal proteins.

The method presented in this study relies heavily on the relationship between the proportion of extensively raised chickens and pigs and the GDP per capita. Obviously, GDP alone cannot explain all existing variability in production systems. Different countries with similar GDP per capita may have different levels of intensive production depending on their history of agricultural development, political economies and indeed cultural factors, and these differences are not taken into account. However, measurement errors in GDP, input *P*_*ext*_ data, or ignoring important factors influencing the proportion of extensively raised chicken and pigs would essentially affect country and province level estimates. In the spatial prediction, these may influence how countries and Chinese provinces contrast in relation to each other, but they will not propagate to the within-unit spatial distribution of extensively and intensively raised livestock, which is more heavily dependent on the distribution of the rural population. This leads on to another simplifying assumption made in this study: extensively raised chickens and pigs were distributed equally among the rural population. Firstly, the rural-urban divide is a dichotomous division that does not capture the gradual change in likelihood of people raising chickens or pig, moving away from city centres. More specifically, the peri-urban areas where mixtures of agricultural and non-agricultural activities occur are not explicitly accounted for. This is apparent from Fig 6, where extensively raised chickens (a) were overestimated in the area surrounding Bangkok compared to the observed distribution based on local census (c). In addition, in rich countries, the rural population can hardly be equated to agricultural population. One way to address this problem would be to develop an agricultural population map, at the pixel level, that decreases with proximity to city centres. This could then be used to assign and thus distribute extensively raised chickens and pigs. However, despite these limitations, the relatively high correlation between observed and predicted proportions of extensively raised chicken and pigs, and the validation made with the Thailand data set suggest that the method effectively captures the main differences between the distributions of extensively and intensively raised chickens and pigs. Fine-scale data on the relative distribution of extensive and intensive production was only available for Thailand, which is the only area where the method has been qualitatively validated, but follow-up investigations will aim to identify complementary data sources in different countries with different socio-economic and environmental conditions.

On a final note, medium and long-term projections of GDP are made by various institutions such as the International Monetary Fund (IMF) and these, in combination with projections of population growth and urbanization rates, could be used to make projections of future distributions of extensive versus intensive production of monogastric livestock species, and to predict trajectories of intensification in different regions of the World. These would ideally also include some spatial clustering algorithms to account for the geographical concentration of production that is widely observed.

The distribution maps provided in this paper are available for download and visualization from the Livestock Geo-Wiki (http://livestock.geo-wiki.org). These distribution data, and possible future projections, can be used for epidemiolocal risk modeling [40], for assessment of antimicrobial usage in poultry and pig production systems [12], and for many other environmental, social and economic assessments of the impacts of intensification monogastric livestock production.

## Supporting Information

S1 Fig Map of the countries where data on the proportion of extensively raised chicken (top) and pigs (bottom) were found through data mining (blue: country with data, grey: country with no data). (TIF)
