# Supplementary figures and images for: Income Disparities and the Global Distribution of Intensively Farmed Chicken and Pigs
Source: PLoS One. 2015 Jul 31;10(7):e0133381. doi: 10.1371/journal.pone.0133381 (PMC4521704; doi:10.1371/journal.pone.0133381)

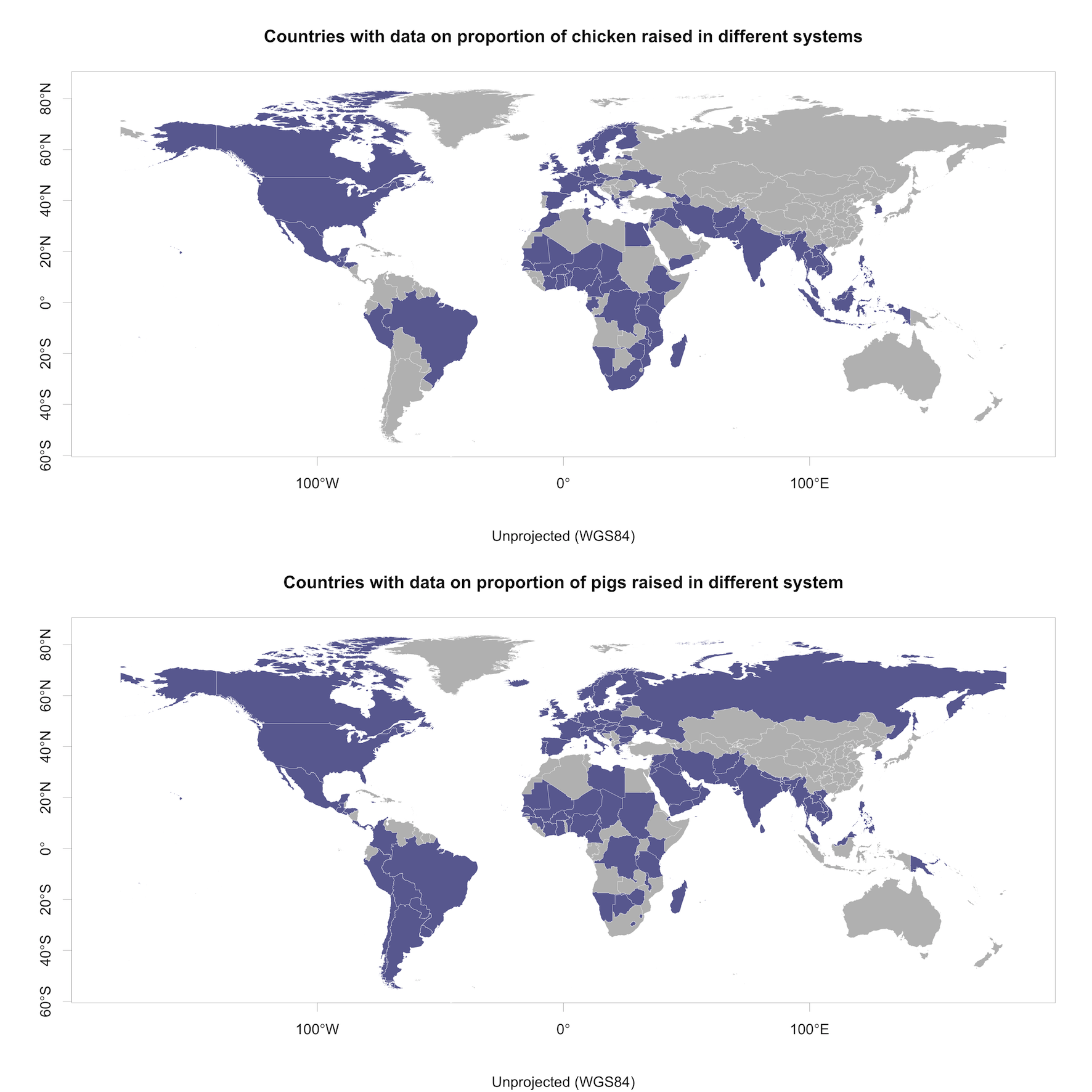

Supplement: S1 Fig — (TIF) [file pone.0133381.s001.tif]
